# *SLC6A4* Repeat and Single-Nucleotide Polymorphisms Are Associated With Depression and Rest Tremor in Parkinson's Disease: An Exploratory Study

**DOI:** 10.3389/fneur.2019.00333

**Published:** 2019-04-09

**Authors:** Jian-Yong Wang, Qian-Ya Fan, Jia-Hui He, Shi-Guo Zhu, Chen-Ping Huang, Xiong Zhang, Jian-Hong Zhu

**Affiliations:** ^1^Department of Neurology, The First People's Hospital of Jiande, Hangzhou, China; ^2^Department of Geriatrics and Neurology, The Second Affiliated Hospital and Yuying Children's Hospital, Wenzhou Medical University, Wenzhou, China; ^3^Department of Preventive Medicine, School of Public Health, Wenzhou Medical University, Wenzhou, China

**Keywords:** Parkinson's disease, serotonin reuptake transporter, SLC6A4, depression, tremor

## Abstract

**Introduction:** Level of serotonin is mainly regulated by the serotonin reuptake transporter encoded by *SLC6A4*. The promoter region of *SLC6A4* bears a repeat polymorphism 5-HTTLPR and a single nucleotide polymorphism rs25531. We have previously studied the association between these two variants and sporadic PD. The objective of the current study was to determine whether the *SLC6A4* polymorphisms were associated with key motor and non-motor symptoms of PD.

**Methods:** A total of 370 PD patients of Han Chinese were included. Associations between the *SLC6A4* polymorphisms and PD symptoms including depression, intellectual impairment, tremor and rigidity were analyzed.

**Results:** 5-HTTLPR was associated with depression in PD patients and presence of the LL genotype was protective against the depression risk. The rs25531 was associated with rest tremor in PD and the A allele serves as a recessive risk allele. No associations were found in the two polymorphisms with respect to intellectual impairment and rigidity in the cohort.

**Conclusion:** The current study reveals two PD symptoms associated with *SLC6A4* polymorphisms, and provides new insight into how serotonergic system genetically participates in the symptomatic progression of PD. Further study is warranted in additional populations.

## Introduction

Parkinson's disease (PD) is a common neurodegenerative disease with bradykinesia, rigidity, and rest tremor as its cardinal motor manifestations (1). Non-motor symptoms such as depression, cognitive problems, sleep disorders, and gastrointestinal disturbance have a great impact on life quality in PD patients (2, 3), and are usually present in PD before the onset of motor signs (4).

A hallmark of PD pathology is degeneration of dopaminergic neurons in the substantia nigra, leading to reduction of dopamine output to the striatum (5). In addition to dopamine, dysregulation of serotonin, which is a neurotransmitter linked with cognition and emotional states, also plays an important role in PD progression (6). The serotonin level is mainly regulated by the serotonin reuptake transporter (5-HTT) encoded by the gene *SLC6A4*. There are two polymorphisms in its promoter region, including a repeat polymorphism 5-HTTLPR and a single nucleotide polymorphism rs25531 located within 5-HTTLPR. 5-HTTLPR consists of 14-repeat and 16-repeat variants, as well as several rare variants such as 15-, 19-, 20-, or 22- repeat. The 14-repeat and 16-repeat elements are denoted as the long (L) allele and the short (S) allele, respectively (7). Both 5-HTTLPR and rs25531 have been suggested in regulation of the 5-HTT expression, collectively or separately (8–10).

We have previously studied the association between sporadic PD and *SLC6A4* polymorphisms in a cohort of 504 patients and 504 controls (7). The objective of the current study was to determine whether the *SLC6A4* polymorphisms were associated with key symptoms of PD. Rest tremor and rigidity are two of the major motor symptoms, while bradykinesia is considered as required for PD diagnosis. Postural instability is typically a feature of more advanced stage (4). Non-motor symptoms include depression, intellectual impairment, thought disorder and motivation deficit. Thought disorder also often appears in the advanced stage (4), while motivation deficit is usually mixed with depression (11). Thus, to test the above hypothesis, motor symptoms including rest tremor and rigidity, and non-motor symptoms including depression and intellectual impairment were characterized and analyzed. Not all the patients of the previous cohort (7) were included in the current study because of the availability and exclusion criteria. Additional participants were then recruited.

## Methods

### Patients

A total of 370 PD patients (188 men and 182 women) of Han Chinese ethnicity were included in this study. The median age at onset was 64 (interquartile range, 56–72). The patients were diagnosed by two movement disorder neurologists according to the UK Parkinson‘s Disease Society Brain Bank Criteria (12). Patients with a family history of PD or with secondary and atypical parkinsonism were excluded. All subjects participating in the study signed written informed consents. This study was approved by the Ethics Committee of The Second Affiliated Hospital and Yuying Children's Hospital, Wenzhou Medical University.

### Symptom Evaluation

The non-motor and motor symptoms were assessed with Unified Parkinson's Disease Rating Scale (UPDRS) by face to face interview and physical examination. Patients were assessed in the practically defined OFF medication state (13). Patients taking antidepressants were excluded. The disease duration was used as an indicator of PD severity. Patients with UPDRS scores of the targeted items at or higher than 2 were classified into the corresponding group of the symptoms as reported previously (14).

### Genotyping

Genomic DNA was isolated from peripheral blood using a DNA extraction kit (Tiangen, Beiing, China). The 5-HTTLPR and rs25531 genotypes were determined by polymerase chain reaction-restriction fragment length polymorphism with restriction endonuclease HpaII (New England BioLabs, Beverly, MA) as previously described (7). The primer pair was 5′-TCC TCC GCT TTG GCG CCT CTT CC-3′ and 5′-TGG GGG TTG CAG GGG AGA TCC TG-3′.

### Statistical Analysis

Data were analyzed using the statistical package of Predictive Analytics Software 18.0 (PASW, version 18.0) for windows. Kolmogorov-Smirnov test was used for normality test. Differences in age at onset and disease duration between PD subgroups were assessed using Mann-Whitney Test. Differences in gender and genotype frequencies were assessed using Chi square test. Multivariate analysis was performed by binary logistic regression model using stepwise forwards method with gender, age at onset, disease duration, 5-HTTLPR, rs25531, and the interaction between the two polymorphisms as covariates. A two-tailed *P* < 0.05 was considered statistically significant.

## Results

### Analysis of *SLC6A4* Polymorphisms With PD Depression and Intellectual Impairment

Two non-motor symptoms including depression and intellectual impairment were analyzed in association with 5-HTTLPR and rs25531 variants of *SLC6A4* in the PD patients. A significant difference (*P* = 0.023) in 5-HTTLPR was detected between PD patients with and without depression (Table 1). Further multivariate analysis confirmed that 5-HTTLPR was associated with PD depression (*P* = 0.029). Patients carrying the LL genotype had reduced risk toward depression in comparison with the major SS carriers (*P* = 0.01, OR 0.298, 95% CI 0.118–0.749; Table 2). In contrast, no significant difference was found in the variant rs25531 with regard to depression. Neither 5-HTTLPR nor rs25531 was found in association with intellectual impairment in the PD patients (Supplemental Tables S1, S2). But age at onset and disease duration were independent risk factors for intellectual impairment (*P* = 0.013, OR 1.025, 95% CI 1.005–1.045 and *P* = 0.01, OR 1.079, 95% CI 1.018–1.144, respectively; Supplemental Table S2).

**Table 1:** Polymorphic analysis of PD patients with or without depression.

		**PD with depression (*n* = 199)**	**PD without depression (*n* = 171)**	***P***
Men, n (%)		95 (47.7)	93 (54.4)	0.202^a^
Age at onset, year (IR)		65 (57–72)	64 (55–72)	0.379^b^
Duration, year (IR)		1 (0–5)	1 (0–3)	0.246^b^
5-HTTLPR	SS, *n* (%)	119 (59.8)	86 (50.3)	0.023^a^
	SL, *n* (%)	58 (29.1)	60 (35.1)	
	LL, *n* (%)	7 (3.5)	17 (9.9)	
	Rare, *n* (%)	15 (7.5)	8 (4.7)	
rs25531	AA, *n* (%)	158 (79.4)	128 (74.9)	0.361^a^
	AG, *n* (%)	40 (20.1)	40 (23.4)	
	GG, *n* (%)	1 (0.5)	3 (1.8)	

*IR, interquartile range; PD, Parkinson's disease*.

a *Analyzed by Chi square test*.

b *Analyzed by Mann-Whitney test*.

**Table 2:** Multivariate risk analysis for PD with depression^a^.

**Factors**	**Genotype**	**B**	***P***	**OR**	**95% CI**	
					**Lower**	**Upper**
5-HTTLPR			0.029			
	SL vs. SS	−0.359	0.122	0.699	0.443	1.101
	LL vs. SS	−1.212	0.010	0.298	0.118	0.749
	Rare vs. SS	0.304	0.509	1.355	0.550	3.339
Constant		0.325	0.022	1.384		

*CI, confidence interval; OR, odds ratio; PD, Parkinson's disease*.

a *Analyzed by binary logistic regression with gender, age at onset, disease duration, 5-HTTLPR, rs25531, and the interaction between the two polymorphisms as covariates*.

### Analysis of *SLC6A4* Polymorphisms With Tremor and Rigidity in PD Patients

Motor symptoms of tremor and rigidity were analyzed to understand their association with *SLC6A4* polymorphisms. Results showed that genotypic distribution of rs25531, but not that of 5-HTTLPR, was significantly different between PD patients with and without tremor (*P* = 0.014; Table 3). Multivariate analysis confirmed that rs25531 was an independent risk factor for tremor in PD patients (*P* = 0.03) with the GG genotype being protective compared to the AA (*P* = 0.032, OR 0.113, 95% CI 0.015–0.831; Table 4). Further analysis showed that PD patients carrying GG and AG conferred lower risk toward tremor than the AA carriers (*P* = 0.031, OR 0.480, 95% CI 0.246–0.934; Table 4), suggesting that A is a recessive risk allele. No significant difference was found in both 5-HTTLPR and rs25531 with regard to rigidity in the patients (Supplemental Tables S3, S4).

**Table 3:** Polymorphic analysis of PD patients with or without tremor.

		**PD with tremor (*n* = 325)**	**PD without tremor (*n* = 45)**	***P***
Men, n (%)		165 (50.8)	23 (51.1)	0.966^a^
Age at onset, year (IR)		64 (56–72)	64 (53.5–72)	0.629^b^
Duration, year (IR)		1 (0–4)	1 (0–4)	0.278^b^
5-HTTLPR	SS, *n* (%)	181 (55.7)	24 (53.3)	0.887^a^
	SL, *n* (%)	104 (32.0)	14 (31.1)	
	LL, *n* (%)	21 (6.5)	3 (6.7)	
	Rare, *n* (%)	19 (5.8)	4 (8.9)	
rs25531	AA, *n* (%)	257 (79.1)	29 (64.4)	0.014^a^
	AG, *n* (%)	66 (20.3)	14 (31.1)	
	GG, *n* (%)	2 (0.6)	2 (4.4)	

*IR, interquartile range; PD, Parkinson's disease*.

a *Analyzed by Chi square test*.

b *Analyzed by Mann-Whitney test*.

**Table 4:** Multivariate risk analysis for PD with tremor^a^.

**Factors**	**Genotype**	**B**	***P***	**OR**	**95% CI**	
					**Lower**	**Upper**
rs25531			0.030			
	AG vs. AA	−0.631	0.074	0.532	0.266	1.064
	GG vs. AA	−2.182	0.032	0.113	0.015	0.831
Constant		2.182	< 0.001	8.862		
rs25531	GG+AG vs. AA	−0.735	0.031	0.480	0.246	0.934
Constant		2.182	< 0.001	8.862		

*CI, confidence interval; OR, odds ratio; PD, Parkinson's disease*.

a *Analyzed by binary logistic regression with gender, age at onset, disease duration, 5-HTTLPR, rs25531, and the interaction between the two polymorphisms as covariates*.

## Discussion

The symptomatology of PD is heterogeneous. Both genetic and environmental factors are believed to be involved in its progression. By analyzing associations of the *SLC6A4* polymorphisms with depression, intellectual impairment, tremor and rigidity, we demonstrate in a currently largest PD cohort that 5-HTTLPR is linked with PD depression and rs25531 is associated with rest tremor.

Extensive evidence have indicated that the 5-HTTLPR is associated with depression risk (15–19). A complicated regulatory mechanism may be involved as suggested in a gene-by-environment study where the association is only present in condition of stressful life events (15). The PD-associated depression exhibits lesser self-punitive behavior and greater anxiety (20), and the depressive symptoms often fluctuate with motor fluctuations (21). As earlier noted, 5-HTTLPR is a functional repeat polymorphism and the S allele leads to reduced 5-HTT expression and serotonin uptake compared to the L allele (22). Different from Caucasians whose SS genotype accounts for 22%, the LL genotype is instead the minor form in Asians and as in our population (23). Our results show that 5-HTTLPR is associated with depression in PD patients with the minor LL genotype serving a protective form. In comparison, four previous studies in smaller sample size displayed mixed results. Two of them in a sample size of 72 and 32, respectively, displayed similar results that patients carrying the LL genotype of 5-HTTLPR were less depressed than the SS and/or SL carriers (24, 25). Nonetheless, other two studies with 89 and 306 patients, respectively, did not find association between 5-HTTLPR and PD depression (26, 27). As a note, some of the PD patients were taking antidepressant medication when their clinical symptoms were evaluated in these two studies (26, 27). Such cases were excluded in the current study, while no relevant information was disclosed in the former two studies (24, 25).

Highlighted should be that we firstly report an association between rs25531 and PD tremor with its A allele as a recessive risk allele. Interestingly, rest tremor is indeed considered to be more likely related to serotonin dysfunction than to dopamine deficiency as suggested by post-mortem and SERT imaging results (28–30). Serotoninergic antidepressant drugs have long been proved effective in alleviating functional tremor (31). However, the results of the antidepressants for PD-associated tremor appear mixed. For instance, a study in 25 PD patients reported that mirtazapine might alleviate rest tremor (32). But two duloxetine-treated PD patients showed an exacerbation of tremor in a multicenter randomized study (33), which however was not seen in another study of patients treated with duloxetine (34). Moreover, an animal study showed that administration of fluoxetine exacerbated oral tremor (35). Similar to 5-HTTLPR, rs25531 is also a functional polymorphism with the G allele leading to reduced efficiency of HTT transcription (9). It has been reported that 5-HTTLPR affects brain cortical development and the functionality of related brain circuitries, particularly between perigenual cingulate and amygdala, thus contributing its genetic susceptibility to depression (36). Nonetheless, it remains to be further explored how rs25531 is mechanistically involved in the modulation of PD tremor. At the least, our results provide further evidence linking serotonin dysfunction to the occurrence of rest tremor in PD.

The *SLC6A4* polymorphisms are not associated with intellectual impairment and rigidity in the patients. However, our results suggest that the cognitive disturbance is related to disease duration and age at onset. While the role of age at onset appears disputable, it is widely recognized that disease duration is associated with dementia in PD (37–39). The current study was initially designed for PD association study (7), and not specifically aimed for the non-motor symptoms. Thus, the main method used was the UPDRS score assessment, instead of a specific scale for cognitive impairment or depression, which otherwise should have been more informative. Also, the number of certain genotypes is limited such as the GG of rs25531. To consolidate our finding, we analyzed the association by comparing GG+AG vs. AA and the results showed similar. Nonetheless, future investigations are warranted to confirm our findings in additional populations.

In conclusion, the current study suggests a role of *SLC6A4* polymorphisms in risk toward PD motor and non-motor symptoms. Specifically, our results are in support that 5-HTTLPR modulates PD depression, and for the first time show that rs25531 is associated with rest tremor in PD, thus providing new insight into how serotonergic system genetically participates in the symptomatic progression of PD.

## Author Contributions

J-YW and J-HZ conceived and designed the study. Q-YF, J-YW, XZ, J-HH, and S-GZ collected samples, acquired and analyzed the data. C-PH provided statistical support and analysis. J-YW and J-HZ interpreted the results and drafted the manuscript. XZ and J-HZ supervised the study. All authors have read, revised, and approved the final version of the manuscript.

### Conflict of Interest Statement

The authors declare that the research was conducted in the absence of any commercial or financial relationships that could be construed as a potential conflict of interest.
