# Supplementary material for: SLC6A4 Repeat and Single-Nucleotide Polymorphisms Are Associated With Depression and Rest Tremor in Parkinson's Disease: An Exploratory Study
Source: Front Neurol. 2019 Apr 9;10:333. doi: 10.3389/fneur.2019.00333 (PMC6465511; doi:10.3389/fneur.2019.00333)
Supplement: Supplementary file 1 [file Data_Sheet_1.docx]

***Supplementary Material*:**

***SLC6A4* repeat and single-nucleotide polymorphisms are associated with depression and rest tremor in Parkinson’s disease**

Jian-Yong Wang^a,b,c,1^, Qian-Ya Fan^a,1^, Jia-Hui He^b^, Shi-Guo Zhu^b^, Chen-Ping Huang^c^, Xiong Zhang^b,*^, Jian-Hong Zhu^b,c,*^

* Correspondence：

Jian-Hong Zhu:

jhzhu@wmu.edu.cn

Xiong Zhang:

zhangxiong98@gmail.com

Supplemental Table S1. Polymorphic analysis of PD patients with or without intellectual impairment

|  | | PD with intellectual impairment (n = 164) | PD without intellectual impairment (n = 206) | *P* |
| --- | --- | --- | --- | --- |
| Men, n (%)  Age at onset, year (IR)  Duration, year (IR) | | 90 (54.9) | 98 (47.6) | 0.163^a^ |
|  |  | 65 (57-72) | 63 (55-71) | 0.142^b^ |
|  |  | 2 (0-5) | 1 (0-3) | 0.044^b^ |
| 5-HTTLPR | SS, n (%) | 90 (54.9) | 115 (55.8) | 0.985^a^ |
|  | SL, n (%) | 52 (31.7) | 66 (32.0) |  |
|  | LL, n (%) | 11 (6.7) | 13 (6.3) |  |
|  | rare, n (%) | 11 (6.7) | 12 (5.8) |  |
| rs25531 | AA, n (%) | 125 (76.2) | 161 (78.2) | 0.614^a^ |
|  | AG, n (%) | 38 (23.2) | 42 (20.4) |  |
|  | GG, n (%) | 1 (0.6) | 3 (1.5) |  |

IR, interquartile range; PD, Parkinson’s disease

^a^ Analyzed by Chi square test

^b^ Analyzed by Mann-Whitney Test

Supplemental Table S2. Multivariate risk analysis for PD with intellectual impairment^a^

| Factors | B | *P* | OR | 95% CI | |
| --- | --- | --- | --- | --- | --- |
|  |  |  |  | Lower | Upper |
| Age at onset | 0.025 | 0.013 | 1.025 | 1.005 | 1.045 |
| Duration | 0.076 | 0.010 | 1.079 | 1.018 | 1.144 |
| Constant | -2.014 | 0.003 | 0.133 |  |  |

CI, confidence interval; OR, odds ratio; PD, Parkinson’s disease

^a^ Analyzed by binary logistic regression with gender, age at onset, disease duration, 5-HTTLPR, rs25531, and the interaction between the two polymorphisms as covariates.

Supplemental Table S3. Polymorphic analysis of PD patients with or without rigidity

|  | | PD with rigidity (n = 329) | PD without rigidity (n = 41) | *P* |
| --- | --- | --- | --- | --- |
| Men, n (%)  Age at onset, year (IR)  Duration, year (IR) | | 169 (51.4) | 19 (46.3) | 0.544^a^ |
|  |  | 64 (56-72) | 63 (57-73) | 0.662^b^ |
|  |  | 1 (0-4) | 1 (0-4) | 0.992^b^ |
| 5-HTTLPR | SS, n (%) | 187 (56.8) | 18 (43.9) | 0.338^a^ |
|  | SL, n (%) | 100 (30.4) | 18 (43.9) |  |
|  | LL, n (%) | 21 (6.4) | 3 (7.3) |  |
|  | rare, n (%) | 21 (6.4) | 2 (4.9) |  |
| rs25531 | AA, n (%) | 254 (77.2) | 32 (78.0) | 0.777^a^ |
|  | AG, n (%) | 71 (21.6) | 9 (22.0) |  |
|  | GG, n (%) | 4 (1.2) | 0 (0) |  |

IR, interquartile range; PD, Parkinson’s disease

^a^ Analyzed by Chi square test

^b^ Analyzed by Mann-Whitney Test

Supplemental Table S4. Multivariate risk analysis for PD with rigidity^a^

| Factors | B | *P* | OR | 95% CI | |
| --- | --- | --- | --- | --- | --- |
|  |  |  |  | Lower | Upper |
| Constant | 2.082 | <0.001 | 8.024 |  |  |

CI, confidence interval; OR, odds ratio; PD, Parkinson’s disease

^a^ Analyzed by binary logistic regression with gender, age at onset, disease duration, 5-HTTLPR, rs25531, and the interaction between the two polymorphisms as covariates.
